# Effect of Bi_2_O_3_ content on the microstructure and electrical properties of SrBi_2_Nb_2_O_9_ piezoelectric ceramics

**DOI:** 10.1039/c8ra01640b

**Published:** 2018-04-25

**Authors:** Xiaochun He, Ruiqing Chu, Zhijun Xu, Zhongran Yao, Jigong Hao

**Affiliations:** School of Environmental and Materials Engineering, Yantai University Yantai 264005 Shandong China ruiqingchu@sohu.com; College of Materials Science and Engineering, Liaocheng University Liaocheng 252059 Shandong China

## Abstract

Lead-free ceramics, SrBi_2_Nb_2_O_9_–*x*Bi_2_O_3_ (SBN–*x*Bi), with different Bi contents of which the molar ratio, *n*(Sr) : *n*(Bi) : *n*(Nb), is 1 : 2(1 + *x*/2) : 2 (*x* = −0.05, 0.0, 0.05, 0.10), were prepared by conventional solid-state reaction method. The effect of excess bismuth on the crystal structure, microstructure and electrical properties of the ceramics were investigated. A layered perovskite structure without any detectable secondary phase and plate-like morphologies of the grains were clearly observed in all samples. The value of the activation energy suggested that the defects in samples could be related to oxygen vacancies. Excellent electrical properties (*e.g.*, *d*_33_ = 18 pC N^−1^, 2*P*_r_ = 17.8 μC cm^−2^, *ρ*_rd_ = 96.4% and *T*_c_ = 420 °C) were simultaneously obtained in the ceramic where *x* = 0.05. Thermal annealing studies indicated the SBN–*x*Bi ceramics system possessed stable piezoelectric properties, demonstrating that the samples could be promising candidates for high-temperature applications.

## Abstract 2

Lead-free ceramics, SrBi_2_Nb_2_O_9_–*x*Bi_2_O_3_ (SBN–*x*Bi), with different Bi contents of which the molar ratio, *n*(Sr) : *n*(Bi) : *n*(Nb), is 1 : 2(1 + *x*/2) : 2 (*x* = −0.05, 0.0, 0.05, 0.10), were prepared by conventional solid-state reaction method.

## Graphical abstract

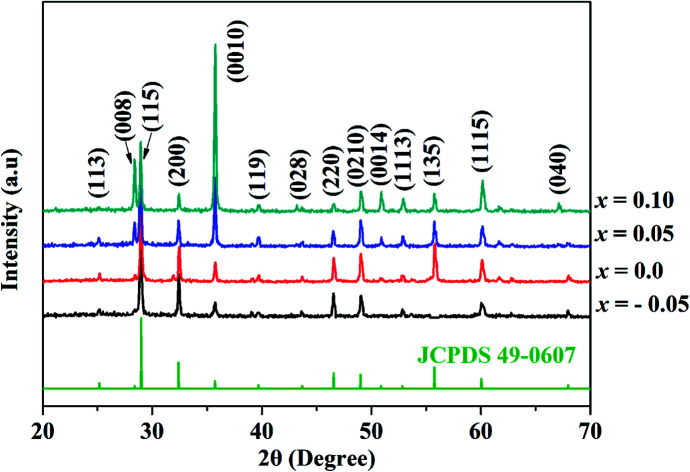

## 1. Introduction

Lead-based ceramics, such as Pb(Zr,Ti)O_3_, have been widely used in applications of the economic industry field, including piezoelectric transformers, sensors and actuators, because of their excellent piezoelectric and ferroelectric properties.^1,2^ However, with the ever-increasing demands relating to environmental concerns, the lead-based ferroelectric and piezoelectric materials are facing several challenges for actual electronic applications due to the high toxicity of lead oxide.^3^ Therefore, it is necessary to develop lead-free compounds for the replacement of lead-based ceramics.

Bismuth layer-structured ferroelectric ceramics (BLSFs), known as the Aurivillius family of oxides, have been considered as one kind of promising lead-free piezoelectrics in high-temperature applications because of their high Curie temperatures, low dielectric loss and low aging rates.^4–6^ The structure of these ferroelectrics consists of [Bi_2_O_2_]^2+^ layers interleaved with a perovskite block of [A_*m*−1_B_*m*_O_3*m*+1_]^2−^ units stacked along the crystallographic *c*-axes, where *m* represents the number of perovskite blocks and the *m* value is generally in the range of 1–5. Additionally, the number of [BO_6_] octahedrals included in the pseudo-perovskite structure is reported to have a close relationship with dielectric and ferroelectric properties.^7,8^ SrBi_2_Nb_2_O_9_ (abbreviated as SBN), as the *m* = 2 member of the Aurivillius family with Sr and Bi ions at the A sites and Nb ions at the B sites, consists of (Bi_2_O_2_)^2+^ layers and pseudo-perovskite ((SrNb_2_O_7_)^2−^) units with double NbO_6_ octahedral layers.^9^ Among BLSFs, SBN has been extensively studied due to its relatively lower crystallization temperature and it is a promising material for high-temperature piezoelectric applications such as piezoelectric accelerometer, especially in the vibration monitoring system at nuclear power plants, owing to its high Curie temperature of 450 °C and high electrical resistance of 10^6^ Ω cm at 375 °C.^10^ However, as other BLSFs compounds, the remanent polarization and piezoelectric activities of SBN ceramics are relatively low due to two-dimensional orientation restriction in rotation of spontaneous polarization, which limits its applicability in piezoelectric devices requiring high temperatures.^11^

Many studies have focused on enhancing the electrical properties of BLSFs, such as change of the grain size,^12^ control of preferred orientation,^13^ and A and/or B-site substitution.^7,8,10^ In addition, it is well known that highly volatile nature of Bi ions at high-temperature heat treatment will cause a non-stoichiometric composition along with the defects of (V_Bi^3+^_)′′′ and (V_O^2+^_)′′, which could weaken the electrical properties of ceramic because of the large ferroelectricity and better electromechanical properties of the ceramics are attributed to Bi^3+^ ions.^14,15^ Thus, many practices have been performed to qualitatively add excess amounts of raw Bi_2_O_3_ powders to compensate for the potential loss and then affect the electrical properties as reported in Bi-containing functional ceramics and films.^14,16,17^ For instance, Qin *et al.*^18^ reported a CaBi_2_Nb_2_O_9_ + 1 wt% Bi_2_O_3_ ceramic with optimal electrical properties as follows: piezoelectric constant *d*_33_ = 6.4 pC N^−1^, resistivity *ρ* = 2.9 × 10^6^ Ω cm (@500 °C) and Curie temperature *T*_c_ = 940 °C. However, there have been few reports regarding the effect of excess Bi on the structure and electrical properties of SBN ceramics, in spite of the possibility of compositional variation induced by volatilization of bismuth element. In this study, SBN ceramic was selected as a host material and the structural defects of the SBN ceramic were compensated by regulating the Bi_2_O_3_ concentration, to modify the poling process and the electrical properties of ceramics. The optimal Bi_2_O_3_ concentration was investigated and the structure–property relationships and possible mechanism were additional discussed.

## 2. Experimental procedure

Polycrystalline SrBi_2_Nb_2_O_9_–*x*Bi_2_O_3_ (SBN–*x*Bi, *x* = −0.05, 0.0, 0.05, 0.10) ceramics were prepared *via* solid-state reaction method using SrCO_3_ (99%), Nb_2_O_5_ (99.5%) and Bi_2_O_3_ (99.99%). All raw materials were ball milling in a polyethylene with stabilized zirconia balls for 15 h. The mixed powders dried and initially pre-calcined at 800 °C for 2 h. Then, the mixture was milled again for 12 h. The slurries were dried and pressed into pallets of thickness 0.5 mm and 12 mm in diameter. The pallets were sintered at 1100 °C for 3 h. For the electric measurements, disk samples with about 0.3 mm in thickness were used.

The density of the sintered ceramics was measured by means of the Archimedes method. The phase constituent of the sintered samples was determined by X-ray diffraction (XRD) using a Cu Kα radiation (*λ* = 1.54178 Å) (D8 Advance, Bruker Inc., Germany). The surface morphology of the ceramics was observed by scanning electron microscope (SEM) (JSM-6380, Japan). The ferroelectric hysteresis loops were measured through standardized ferroelectric test system (TF2000, Germany). The temperature dependence of dielectric properties and impedance spectroscopy for the samples was performed using a Broadband Dielectric Spectrometer (Novocontrol Germany). The samples were polarized in silicon oil in the range of 150–180 °C for 20 min, and piezoelectric measurements were carried out with a quasi-static *d*_33_-meterYE2730 (SINOCERA, China).

## 3. Results and discussions

**Fig. 1:**
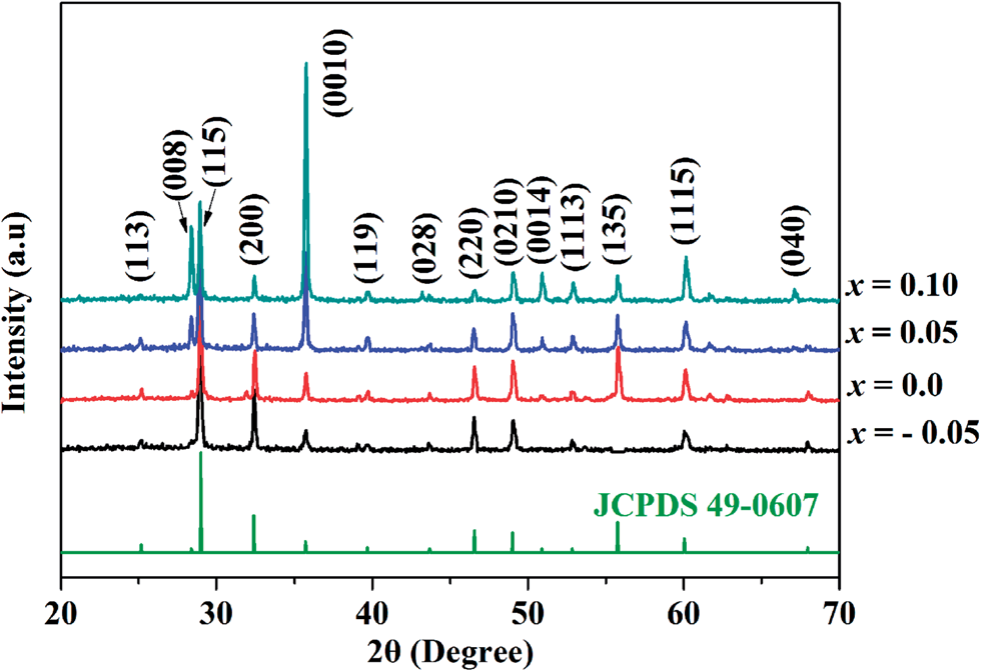
XRD patterns of SBN–*x*Bi ceramics sintered at 1100 °C.

The X-ray diffraction spectra of SBN–*x*Bi ceramics in the 2*θ* range of 20–70°are shown in Fig. 1. It is obvious that all the prominent peaks show the samples possess a single bismuth-layered perovskite structure without any detectable secondary phases, indicating that Bi_2_O_3_ has diffused into SrBi_2_Nb_2_O_9_ lattices to form a new solid solution SBN–*x*Bi. In addition, when the doping content is lower, the highest diffraction peak of SBN–*x*Bi ceramics is (115) orientation, which is in good agreement with the highest diffraction peaks of (112*m* + 1) in BLSFs phase and belongs to orthorhombic structure (JCPDS 49-0607).^10^ However, with the increase of the doping content, the (0010) peak enhanced gradually, and the peak value is much higher than that of (115) peak when the Bi_2_O_3_ content is 0.10, which could be ascribed to that the excess Bi_2_O_3_ can act as a sintering additive affecting the crystal growth and then affecting the crystal orientation of SBN–*x*Bi ceramics. Thus, the variation of crystal orientation could affect the microstructure of SBN–*x*Bi ceramics.

**Fig. 2:**
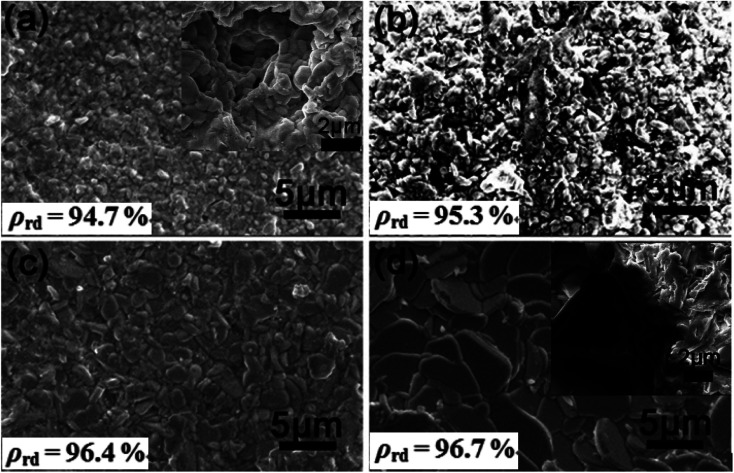
SEM micrographs of SBN–*x*Bi ceramics sintered at 1100 °C: (a) *x* = −0.05 and inset is the images of thermally-etched surface of SBN–(−0.05)Bi ceramic; (b) *x* = 0.0; (c) *x* = 0.05; (d) *x* = 0.1 and inset is the images of thermally-etched surface of SBN–0.1Bi ceramic.

Fig. 2 depicted the SEM micrographs for the nature surface of SBN–*x*Bi ceramics sintered at 1100 °C. It could be clearly found that the grains of all the samples are well-defined and the distinct pores decrease with increasing Bi_2_O_3_ content, indicating the excess Bi^3+^ could effectively promote sintering of the SBN ceramics and suppress the occurrence of defects. In addition, the grain size increases gradually with the increasing Bi_2_O_3_ content, suggesting that the excess Bi_2_O_3_ could acts as a grain growth accelerant and has an evident effect on grain size of SBN ceramics. In order to determine the increased grain size, we polished and etched the surface of the samples, as shown in inset of Fig. 2(a) and (d). It is clear that the grain size of SBN–0.1Bi is much larger than that of SBN–(−0.05)Bi, indicating the grain size of ceramics could be increased gradually with the increasing Bi_2_O_3_ content. Such a behavior was also observed in CaBi_2_Nb_2_O_9_ + *x* wt% Bi_2_O_3_ (ref. 18) and BaBi_4_Ti_4_O_15_ + *x* wt% Bi_2_O_3_.^19^ As shown in Fig. 2, all ceramics have a high relative density *ρ*_rd_ (>94%, Table 1) and the *ρ*_rd_ value of the samples increases slightly with increasing Bi_2_O_3_ content, which could be related to the sintering additive promoting of excess Bi_2_O_3_ and the increased grain size: with increasing grain growth, the number of pores was found to decrease, which is beneficial for promoting electrical properties. Moreover, the strongly anisotropic and plate-like morphologies of SBN–*x*Bi samples can be obtained as the Bi_2_O_3_ content up to 0.05 and 0.1, which was typical structure of the ceramic materials based on Aurivillius compounds.^9^

**Table 1:** Remanent polarization (2*P*_r_), coercive field (*E*_c_), Curie temperature (*T*_c_), activation energy (*E*_a_), room density (*ρ*_rd_), piezoelectric coefficient (*d*_33_) for different compositions; room temperature dielectric constant (*ε*_RT_) and loss (tan *δ*) obtained at 1 MHz

Samples (*x*)	*ε*_RT_	tan *δ* (%)	*T*_c_ (°C)	*E*_a_ (eV)	2*P*_r_ (μC cm^−2^)	*E*_c_ (kV cm^−1^)	*d*_33_ (pC N^−1^)	*ρ*_rd_ (%)
−0.05	171	0.5	433	0.57	5.9	66	12	94.7
0.0	160	1.7	445	0.57	15.5	55	16	95.3
0.05	152	0.5	420	0.55	17.8	50	18	96.4
0.10	143	0.6	422	0.55	11.5	54	16	96.7

**Fig. 3:**
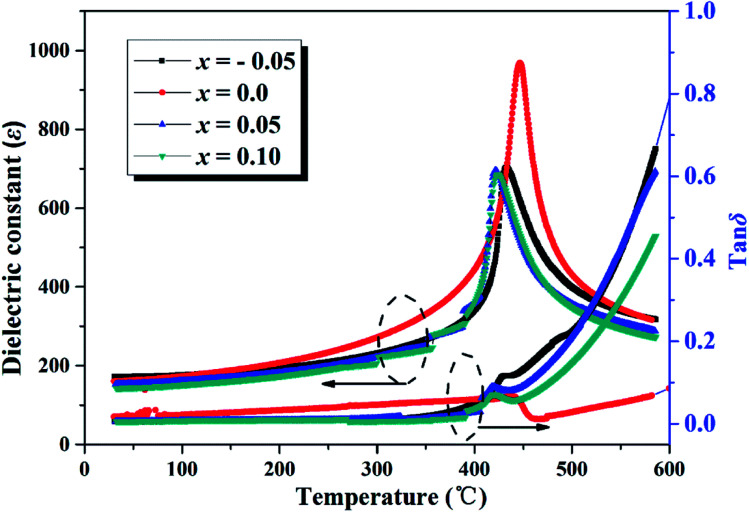
Temperature dependence of dielectric constant (*ε*) and loss (tan *δ*) of SBN–*x*Bi ceramics measured at 1 MHz.

Fig. 3 shows the temperature dependence of dielectric constant (*ε*) and dielectric loss (tan *δ*) of SBN–*x*Bi ceramics at 1 MHz. The principal properties of the SrBi_2_Nb_2_O_9_ + *x*Bi_2_O_3_ ceramics are listed in Table 1. As shown in Fig. 3 and Table 1, only one sharp dielectric peaks, corresponds to the Curie temperature (*T*_c_), appeared when the temperature is higher than 400 °C for all the SBN–*x*Bi ceramics. It is also clear that the dielectric loss values were low and stable when the measurement temperature is below 400 °C, which is of great importance for high-temperature device applications. Interestingly, the dielectric constant and loss of *x* = 0.0 is much larger than other samples, which could be closely related to its small grains as shown in Fig. 2b. Actually, in the BLSF Aurivillius structure, the spontaneous polarization takes place mainly in the *a*–*b* plane and the contribution along the *c* axis is less significant.^18,20^ Therefore, for BLSF polycrystalline ceramics, the grain size strongly affects the electrical properties as reported by Chen *et al.*^21^ In addition, the tan *δ* value significantly increased when the temperature was above 450 °C, which could be related to the space charge carriers induced by the increase of electrical conductivity at high temperatures.^22^

**Fig. 4:**
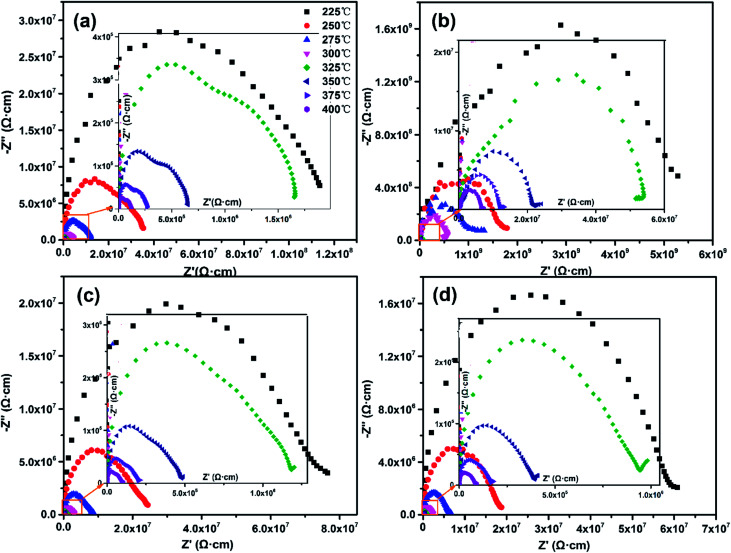
The complex impedance spectra of SBN–*x*Bi ceramics: (a) *x* =−0.05; (b) *x* = 0.0; (c) *x* = 0.05; (d) *x* = 0.1.

Fig. 4 shows the complex impedance spectra of SBN–*x*Bi ceramics in the temperature range of 225 °C to 400 °C at 0.01 Hz to 20 MHz. It is clear that two semicircles at different temperatures exist in SBN–*x*Bi ceramics with *x* = −0.05, 0, 0.05. Semicircles at each temperature can be segregated into two natural electrical components corresponding to the two fitting semicircles in the impedance plots.^23^ In the higher frequency range, the larger semicircle is ascribed to the grain effect (modeled by an equivalent circuit *R*_b_*C*_b_), whereas the grain boundary response (modeled by an equivalent circuit *R*_gb_*C*_gb_) contributes to the smaller semicircle in the lower frequency range.^23,24^ Interestingly, with the increasing of Bi_2_O_3_ contents, only a single semicircle is observed for SBN–*x*Bi at each temperature, and these curves can be fitted to the standard semicircles with the grain (*R*_b_*C*_b_) element, indicating that a single localized relaxation mechanism (grain effect) dominates the impedance in the measured temperature range.^25^ In addition, as shown in Fig. 4, the semicircles shift to small with temperature increasing, indicating the decrement of the resistivity for SBN–*x*Bi ceramics. Finally, the slope of the curves bowed to the real axis (*Z*′), and the depressed semicircle arc were obtained at high temperature, representing the indicative of the presence of both located and nonlocated conduction processes.^26,27^ By extrapolating the low-frequency intercept of the real axis, the resistance could be obtained.^28^

**Fig. 5:**
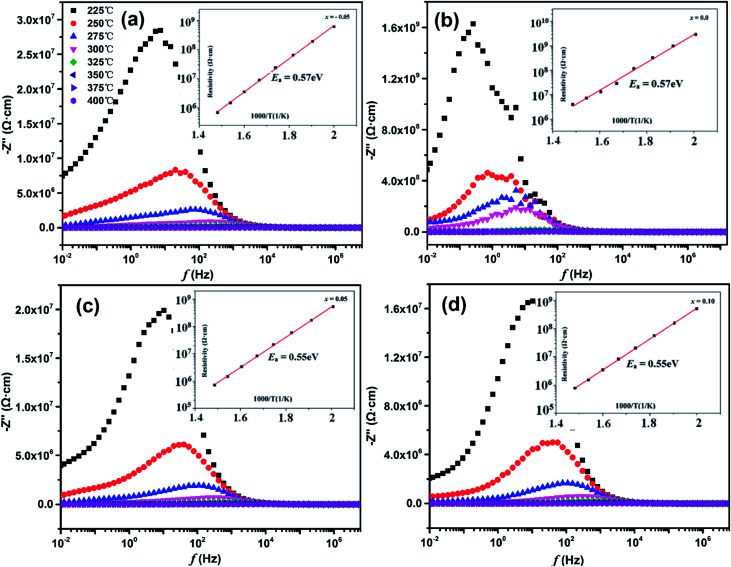
Variation of imaginary part of impedance (*Z*′′) with frequency at different temperatures for different doping concentrations of bismuth. (a) *x* = −0.05; (b) *x* = 0.0; (c) *x* = 0.05; (d) *x* = 0.1. Inset: the temperature dependence of resistivity of SBN–*x*Bi ceramics.

In order to further confirm the resistance of SBN–*x*Bi ceramics, the variation of imaginary part of the impedance (*Z*') with frequency was shown in Fig. 5. One can see that only one peak appeared in the imaginary part for all samples, indicating that only one electrical component contribute to the conductivities of the ceramics, which corresponds to the undistorted Debye-like semicircles.^2,29^ Additionally, it is clear that the peaks were asymmetric and broadened and their positions shifted toward higher frequency side with the temperature increasing, suggesting the presence of electrical processes in the materials with a different relaxation time.^30^

The DC resistivity of the SBN–*x*Bi ceramic as a function of reciprocal temperature was shown in the inset of Fig. 5. It is noted that the resistivity of all the samples are higher than 10^5^ Ω cm even if the temperature reach up to 400 °C, indicating the SBN–*x*Bi ceramics possessed a higher insulation resistivity, which is of necessity for piezoelectric ceramics to ensure a large electric field can be applied during poling without breakdown or excessive charge leakage and thus enhance the electrical properties.^22,31^ Moreover, the behavior of the temperature dependent resistivity follows Arrhenius relationship:

1 *ρ* = *A* exp(−*E*_a_/*k*_B_*T*)

where *A* is a pre-exponential factor constant, *E*_a_ is the activation energy of the mobile charge carriers, *k*_B_ is the Boltzmann constant and *T* is the absolute temperature.^22^ According to Eqn (1), the value of the activation energy *E*_a_ for the SBN–*x*Bi ceramic, calculated by the linear fitting of the data points, was calculated to be 0.55–0.57 eV (Table 1), which were close to the activation energy values of the ionic conductivity by oxygen vacancies in perovskite type ferroelectric oxides.^32–34^

**Fig. 6:**
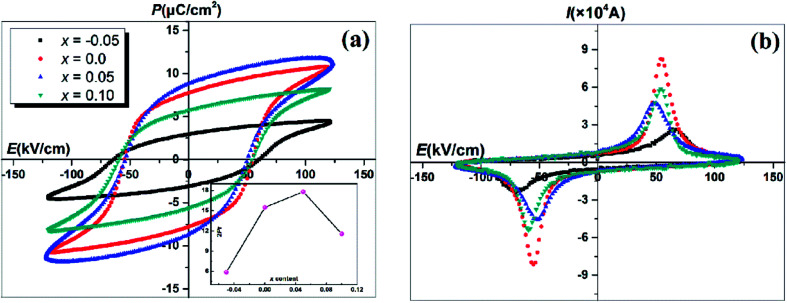
(a) *P*–*E* hysteresis loops of SBN–*x*Bi ceramics with different *x* at 180 °C; the inset shows the detailed information on the response of 2*P*_r_ as a function of *x*; (b) the polarization current curves of the SBN–*x*Bi ceramics.

Fig. 6(a) exhibits the *P*–*E* hysteresis loops of SBN–*x*Bi ceramics measured at 10 Hz and 180 °C under an electric field of 125 kV cm^−1^. Detailed information on the response of the variation of remanent polarization (2*P*_r_) for SBN–*x*Bi ceramics as a function of *x* is provided in inset of Fig. 6(a). It is evident that all of the samples exhibited the typical *P*–*E* loops and the 2*P*_r_ increases at first, then decreases, while the coercive field (*E*_c_) changes slightly with the increase of Bi_2_O_3_ content, which indicates that the Bi_2_O_3_ content plays a significant influence on ferroelectric properties. When the doping content is 0.05, the 2*P*_r_ obtained a maximum value of 17.8 μC cm^−2^ with a relatively low coercive field (50 kV cm^−1^, Table 1), suggesting the polarization state of SBN–*x*Bi ceramics could be enhanced surprisingly within a proper range of Bi_2_O_3_ content. Proper Bi_2_O_3_ content lowered the concentration of oxygen vacancies and improve the chemical stability of the Bi_2_O_2_ layer and perovskite blocks, which is helpful to weaken the influence of domain pinning and result in the increase of the 2*P*_r_.^35^ In addition, the polarization current curves of the SBN–*x*Bi ceramics were collected so that the polarization state could be further realized, as shown in Fig. 6(b). It is clear that only one sharp polarization current peak could be observed when the applied electric field reached *E*_c_, indicating that the ferroelectric domain could be easier to switch when the driving electric field increases to *E*_c_.^36^

**Fig. 7:**
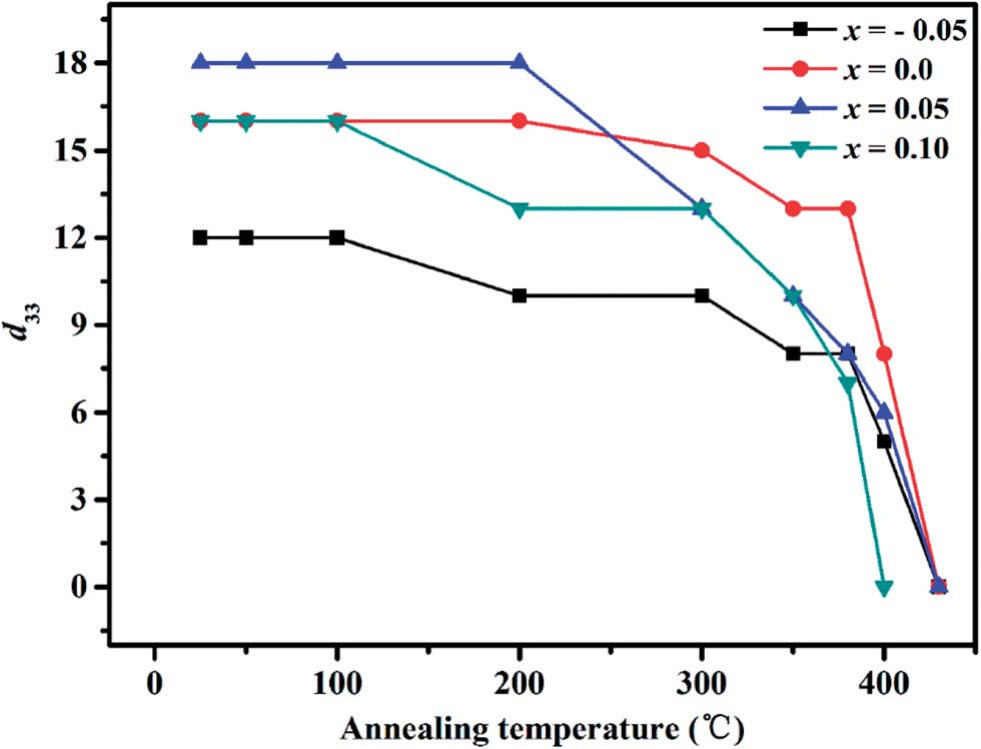
Annealing temperature dependence of piezoelectric coefficient (*d*_33_) for SBN–*x*Bi specimens.

Fig. 7 shows the piezoelectric coefficient (*d*_33_) as a function of operating temperature for SBN–*x*Bi piezoelectric ceramics. It is obvious that the *d*_33_ increased at first and then decreased as the Bi_2_O_3_ content increases at room temperature. The excellent piezoelectric coefficient is founded to be 18 pC N^−1^ when *x* is 0.05, which is higher than that of pure SBN ceramic as shown in Fig. 7 and Table 1. The enhanced piezoelectric activity could be attributed to the enhanced polarizability caused by the appropriate lattice distortion and the decreased amount of oxygen vacancies of the ceramics because of the regulating of Bi_2_O_3_ concentration. Moreover, increasing plate-like grains imply decreased polarization efficiency under high electric field poling condition, as related to the microstructures shown in Fig. 2(d) for the ceramics with *x* = 0.1, leading to the deteriorated piezoelectric properties.^37^ Additionally, the *d*_33_ of all samples decreases rapidly to zero or near zero as the annealing temperature is above *T*_c_, which should be related to the increasing tan *δ* as the temperature increases, as shown in Fig. 3, indicating the nature of the ferro–paraelectric phase transition.^22^ Corresponding to this, the *d*_33_ value of SBN–*x*Bi ceramics remains essentially temperature-independent up to 300 °C, exhibiting the ceramics have a good thermal stability for high temperature applications.

## 4. Conclusions

In this article, we have successfully prepared SrBi_2_Nb_2_O_9_–*x*Bi_2_O_3_ (SBN–*x*Bi) piezoelectric ceramics using conventional solid-state progressing under the same sintering temperature of 1100 °C. The SBN–*x*Bi ceramics presented a typical layered perovskite structure and the morphologies of Aurivillius ceramics show the grains of all the samples are well-defined and the grain size of the ceramics increases with the increase in Bi_2_O_3_ content. The piezoelectric coefficients (*d*_33_) increases up to be 18 pC N^−1^ and the remanent polarization (2*P*_r_) increased up to a value of 17.8 μC cm^−2^ with a relatively low coercive field (50 kV cm^−1^) when *x* is 0.05. As a result, a high *d*_33_, 2*P*_r_ and good thermal stability sample has been attained in the SBN–0.05Bi ceramic. Therefore, such a material system is a potential candidate for high-temperature piezoelectric applications.

## Conflicts of interest

There are no conflicts to declare.

## Supplementary Material
